# Spontaneous Behavior in Noise and Silence: A Possible New Measure to Assess Tinnitus in Guinea Pigs

**DOI:** 10.3389/fneur.2014.00207

**Published:** 2014-10-15

**Authors:** Amarins N. Heeringa, Martijn J. H. Agterberg, Pim van Dijk

**Affiliations:** ^1^Department of Otorhinolaryngology/Head and Neck Surgery, University Medical Center Groningen, University of Groningen, Groningen, Netherlands; ^2^Graduate School of Medical Sciences (Research School of Behavioural and Cognitive Neurosciences), University of Groningen, Groningen, Netherlands; ^3^Department of Otorhinolaryngology, Donders Institute for Brain, Cognition and Behaviour, Radboud University Nijmegen Medical Centre, Nijmegen, Netherlands; ^4^Department of Biophysics, Donders Institute for Brain, Cognition and Behaviour, Radboud University Nijmegen, Nijmegen, Netherlands

**Keywords:** guinea pigs, tinnitus, behavioral model, shuttle box, auditory, spontaneous behavior

## Abstract

This study describes two experiments that were conducted in search for a behavioral paradigm to test for tinnitus in guinea pigs. Conditioning paradigms are available to determine the presence of tinnitus in animals and are based on the assumption that tinnitus impairs their ability to detect silent intervals in continuous noise. Guinea pigs have not been subjected to these paradigms yet; therefore, we investigated whether guinea pigs could be conditioned in the two-way shuttle-box paradigm to respond to silent intervals in noise. Even though guinea pigs could be trained relatively easy to respond to the presence of a noise interval, training guinea pigs to silent intervals in noise was unsuccessful. Instead, it appeared that they became immobile when the continuous stimulus was suddenly stopped. This was confirmed by the next experiment, in which we subjected guinea pigs to alternating intervals of noise and silence with a random duration between 30 and 120 s. Indeed, guinea pigs were significantly longer immobile during silence compared to during noise. By interpreting immobility as a signature of perceiving silence, we hypothesized that the presence of tinnitus would reduce immobility in silence. Therefore, we unilaterally exposed one group of guinea pigs to an 11-kHz tone of 124 dB sound pressure level for 1 h. A subset of the exposed animals was significantly more active in silence, but also more active in noise, as compared to the control group. The increased mobility during silent intervals might represent tinnitus. However, the increased mobility in noise of this group implies that the observed behavior could have derived from, e.g., an overall increase in activity. Therefore, conducting validation experiments is very important before implementing this method as a new screening tool for tinnitus. Follow-up experiments are discussed to further elucidate the origin of the increased mobility in both silence and noise.

## Introduction

In the search for a treatment for tinnitus, animal models have proven to be useful to correlate tinnitus-inducing treatments, such as noise exposure, with neurophysiological changes in the central auditory system (1–3). However, the validity of these animal models critically depends on a behavioral paradigm to test whether an animal perceives tinnitus.

In the last few decades, several behavioral paradigms have been developed, which are all based on the assumption that perception of tinnitus impairs the ability to detect silence (4). Current models can be roughly divided between two categories: (1) conditioning paradigms, in which animals are trained to respond to silent intervals in background noise (5), and (2) startle reflex paradigms, in which the acoustic startle reflex is inhibited by a short silent gap in background noise preceding the startle stimulus (6). In both paradigms, it is determined whether animals are still able to detect a silent interval after a tinnitus-inducing treatment, such as noise exposure or salicylate.

Guinea pigs are frequently used to study neurophysiological changes after tinnitus-inducing treatments [e.g., Ref. (7–10)]. Recent studies, that make use of the startle reflex paradigm, suggest that guinea pigs are indeed able to experience tinnitus (11, 12). However, the startle reflex paradigm needs to be adjusted for optimal assessment of tinnitus in guinea pigs (13). On the contrary, as to our knowledge, conditioning paradigms have not been used to assess tinnitus in guinea pigs yet.

One successful paradigm to condition guinea pigs to acoustic stimuli was presented by Philippens and colleagues (14). This paradigm involved the use of a two-way shuttle box (15). Within a few days of training, guinea pigs learn to shuttle from one compartment to the other upon presentation of a narrowband noise, i.e., the conditioned stimulus (CS). By their shuttle behavior, they avoid an unpleasant stream of air that serves as the unconditioned stimulus (UCS).

The initial aim of the current study was to develop a conditioning model to detect tinnitus in guinea pigs by using the shuttle-box paradigm. Experiment 1 examined the ability of guinea pigs to be trained in the shuttle box to a silent interval in continuous noise. Although training guinea pigs to shuttle at the presence of noise as CS was relatively easy, they could not be trained to shuttle in response to a silent interval in background noise. Instead, it appeared that the behavioral activity of the animals was inhibited during the silent interval. Therefore, we designed another experiment to investigate spontaneous behavior of guinea pigs during silence. In experiment 2 of the present study, guinea pigs were positioned in the shuttle box during alternating intervals of noise and silence. We evaluated the mobility of guinea pigs during silence and noise. This spontaneous behavior might explain the failure to train guinea pigs to silence (experiment 1). Furthermore, in experiment 2, a subgroup of animals was unilaterally exposed for 1 h to an 11-kHz tone of 124 dB sound pressure level (SPL), which has been previously proposed to induce tinnitus in guinea pigs (12). We hypothesized that animals that hear tinnitus are no longer able to experience silence, and will show increased mobility during the silent intervals.

## Materials and Methods

This study includes two experiments. The results of experiment 1 were used to design experiment 2. Both experiments were approved by the Animal Experiment Committee of the University of Groningen in compliance with Dutch law and regulations (DEC-RUG # 6068C and 6068E, respectively).

### Experiment 1: Training to respond to noise and silent intervals

#### Animals

For experiment 1, 10 male adult albino guinea pigs (Dunkin Hartley; Harlan Laboratories, Horst, the Netherlands), weighing between 300 and 360 g at the start of the experiment, were divided over two experimental groups. Group 1 was first trained to noise and subsequently to silent intervals in continuous noise; group 2 was only trained to silent intervals in continuous noise. During the 10-day acclimatization period, the animals were socially housed in the Central Animal Facility of the University Medical Center Groningen on a 12-h light:12-h dark cycle, and were handled every other day. Food and water were provided *ad libitum*. In the housing room, temperature and humidity were kept constant, and background noise from a radio was present at 50 dB(A). One guinea pig was excluded based on a negative Preyer’s reflex at the start of the training (16).

#### The shuttle box

The shuttle box, adapted from Agterberg et al., consisted of two compartments of equal size (23 cm × 23 cm × 23 cm), which were connected by a passage (17). Infrared beams placed on each side of the passage detected shuttling of the guinea pig between compartments. A forceful stream of air (maximum duration 20 s), that was directed downwards into the compartment where the guinea pig was at that moment, and that terminated upon shuttling, served as the UCS. Guinea pigs were trained to avoid the UCS by shuttling from one compartment to the other upon presentation of the CS. The CS was either a band-limited noise (2–19 kHz, 6 dB/octave slope, 78 dB SPL, 15 s) or a silent interval (15 s) in continuous noise (2–19 kHz, 6 dB/octave slope, 78 dB SPL). Note that in the last case, the absence of sound, rather than the noise, served as CS. Acoustic stimuli were presented by two Piezo tweeters (PH8; Velleman) placed above each of the compartments of the shuttle box, respectively. The inter-trial interval had a random duration between 20 and 30 s during training to noise as CS (as described by Agterberg et al.), and a random duration between 60 and 90 s during training to silent intervals as CS (as described by Sansone and Bovet) (17, 18). This behavioral training paradigm has been described previously with respect to detection of acoustic stimuli with different sound levels (17) and with respect to detection of intracochlear electrical stimulation (19). Custom-made LabView software (National Instruments Corp.) generated acoustic stimuli, controlled UCS and CS presentation, and acquired shuttle responses. Background noise in the experimental room was on average 44 dB(A) and was attenuated by the use of a sound-attenuating box, which was placed over the shuttle box.

#### Behavioral training

In preparation for training, guinea pigs were allowed to habituate to the shuttle box for 20 min/day. Prior to training with noise as CS, guinea pigs were simply placed in the shuttle box and habituated in silence, and prior to training with silent intervals, the habituation occurred in the presence of a continuous noise of 78 dB SPL. A shuttle response during the CS was scored as a correct avoidance response (CAR). The first two training sessions consisted of 10 trials. During the remaining sessions, the number of trials per session was increased to 20. In order to prevent excessive exposure to the UCS, sessions were ended when animals failed to demonstrate a CAR for seven consecutive trials. Habituation and training always started on Wednesday and consisted of 5 and 10 working days, respectively. There were no habituation and training sessions during the weekends.

#### Auditory brainstem response recordings

To verify that the guinea pigs were able to hear the acoustic stimuli, hearing thresholds were determined after the last training session by recording auditory brainstem responses (ABRs) to a broadband click of 0.1 ms. Guinea pigs were anesthetized with isoflurane (5% for initiating and 2% for maintenance of the anesthesia) in a mixture of medical air and oxygen. Body temperature was maintained at 38.5°C by a heating pad, and heart rate and blood oxygen saturation were monitored using a pulsoximeter. Recording electrodes were placed subdermally at the vertex and behind each pinna. Signals were generated by a real-time processor (RP2.1, Tucker Davis Technologies Inc.), attenuated (PA5, TDT Inc.), and presented by an electrostatic speaker driver and speaker (ED1 and ES1, respectively; TDT Inc.). The free-field speaker (ES1) was positioned at approximately 2 cm in front of the nose of the animal. ABRs were amplified (EG&G Instruments, 5113 pre-amp), recorded by a second real-time processor, and saved on a PC using BioSigRP software (TDT Inc.). Stimulus presentation level started at 97 dB SPL and decreased with steps of 10 dB. ABRs were averaged over 1000 presentations. Thresholds were considered the lowest stimulus level to which distinct ABR waves were observed.

### Experiment 2: Spontaneous behavior in noise and in silence

#### Animals

For experiment 2, 15 guinea pigs, weighing between 376 and 488 g at the start of the experiment, were used. The guinea pigs were divided over two groups: an experimental group (*n* = 10) that was exposed to a loud tone and a control group (*n* = 5) that was sham-exposed. The experimental group was larger because previous studies demonstrated that unilateral overexposure leads to tinnitus in only a subset of animals (11, 20). (Sham-) exposure was applied in the afternoon of day 1 and spontaneous behavior to noise and silence was assessed in the morning of the following day. ABRs were recorded immediately before and after (sham-) exposure, and after behavioral testing (Figure 2). Housing conditions were similar to conditions in experiment 1.

#### Auditory brainstem response

Auditory brainstem responses for experiment 2 were acquired with the same equipment as used in experiment 1 (see Auditory Brainstem Response Recordings), but were measured for both ears separately. The free-field speaker was positioned against the pinna of the ipsilateral ear and the contralateral ear was plugged with wax (ohropax; OTC Medical BV). ABRs were recorded for responses to 0.1 ms clicks and to pure tones of 3, 6, 11, 15, and 22 kHz (3 ms duration, 0.2 ms cos^2^ ramps). Stimuli were calibrated using a measuring microphone (Bruël & Kjær; type 2670) and amplifier (B&K; type 2610). ABR thresholds were evaluated by an observer that was blind for stimuli and treatments.

#### Sound exposure

Anesthesia and monitoring of the animals during sound exposure were as described in section “Auditory brainstem response recordings” of Experiment 1. The cone of a Piezo tweeter was removed and a custom-made funnel was attached to the speaker. The narrow end of the funnel was positioned inside the ipsilateral ear, and the contralateral ear was plugged with wax to reduce air-conduction of the trauma stimulus. A continuous 11-kHz tone of 124 dB SPL was presented for 1 h to the anesthetized animal. The stimulus was designed in RPvdsEx (TDT, Inc.), generated by RP2.1 (TDT, Inc.) and amplified by a Philips amplifier (Philips PM 5170). The stimulus was calibrated at the narrow end of the funnel by a measuring microphone (B&K; type 2670) and amplifier (B&K; type 2610). The sham-exposed group was treated as the experimental group without the stimulus being presented by the speaker.

#### Testing of behavioral activity in noise and silence

Spontaneous behavior of guinea pigs in noise and silence was studied in the shuttle box. The stimulus [designed by custom-made programs in MatLab (2010b, Mathworks)], consisted of alternating noise (band-limited between 2 and 19 kHz, 88 dB SPL) and silence intervals. Noise and silence intervals had durations equal to 30, 60, 90, and 120 s. The intervals were presented in a quasi-random order. However, each session started with a noise interval. Every noise and silence interval was presented twice; hence, the total duration of behavioral testing was 20 min. For each animal, the order of durations of the intervals within the stimulus was individually randomized. Examples of the above-described stimulus can be found in Figure 4. The spontaneous behavior of the guinea pig and the acoustic stimulus were recorded with a smart phone (iPhone 4S, Apple Inc.), that was positioned approximately 1 m above the shuttle box.

Video recordings were muted and analyzed (VLC media player, version 1.1.5) at 0.33 times the rate by an observer blind for the treatment of the animal. Spontaneous behavior was scored for every second as either immobile, moving, walking, or shuttling. Moving refers to movements that did not involve a change of location, e.g., movements of the head. Walking refers to relocation of at least one of the paws. In shuttling, the animal moved from one compartment of the shuttle box to the other, i.e., a shuttle response. One shuttle response counted for 1 s. If an animal showed two types of behavior within 1 s, the most active type of behavior was scored for. Thus, immobility was only scored when the animal was sitting still for the entire second. In the analyses on group level, immobility (in percentage of time) was chosen as the main outcome measure.

### Statistics

Statistical analyses were performed using repeated-measures analysis of variance (RM-ANOVA) and Student’s *T*-test, as appropriate (IBM SPSS Statistics; Version 19). Significance was determined at *p* < 0.05. Data in figures display mean ± SEM.

## Results

### Experiment 1: Training to respond to noise and silence intervals

Auditory brainstem responses to a free-field broadband click revealed that hearing thresholds ranged from 26 to 47 dB SPL across animals. This indicated that all animals could hear the noise of 78 dB SPL, which was either presented as a CS or as a continuous noise with a silent interval.

**Figure 1:**
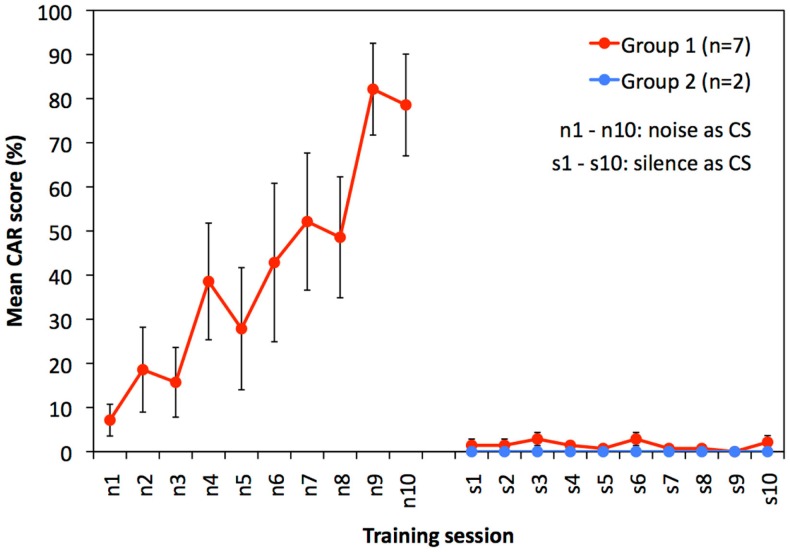
**Results of experiment 1: training to respond to noise and silent intervals are shown**. Mean (±SEM) CAR scores of guinea pigs of group 1 trained to noise (*n* = 7, red circles, sessions n1–n10) and subsequently trained to silence (sessions s1–s10), and of guinea pigs of group 2 (*n* = 2, blue circles) trained only to silence (session s1–s10). RM-ANOVA revealed that performance of group 1 increased during sessions n1–n10 (*F* = 9.048, *p* < 0.001). In contrast, the animals did not show any performance increase during training to silence (s1–s10 of group 1 and 2 combined, RM-ANOVA: *F* = 0.696, *p* = 0.585).

Within 10 training sessions to noise as CS (n1–n10), guinea pigs of group 1 gradually increased their mean performance from <10% CAR in session one (n1) to approximately 80% CAR in session 10 (n10). This is indicative of a learning effect over time (Figure 1, red curve, sessions n1–n10; RM-ANOVA: *F* = 9.048, *p* < 0.001).

Both experimental groups were tested for their ability to condition to a silent interval in continuous noise as CS. Animals in group 1, who were previously trained to noise, showed no scores above 10% CAR in any training session (Figure 1, red curve, sessions s1–s10). Guinea pigs of group 2 that were naïve for training in the shuttle box had no scores above 0% CAR in any of the 10 training sessions (Figure 1, blue curve, session s1–s10).

### Experiment 2: Spontaneous behavior in noise and in silence

**Figure 2:**
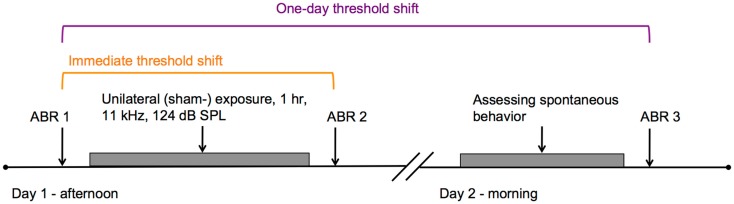
**Timeline of experiment 2 is shown**. In the afternoon of day 1, guinea pigs were either unilaterally exposed to an 11-kHz pure tone of 124 dB SPL, or sham-exposed for 1 h. ABRs were measured immediately before and after the (sham-) exposure, indicated by ABR 1 and ABR 2, respectively. The difference between ABR 1 and ABR 2 was considered as the immediate threshold shift (in orange). In the morning of the subsequent day, behavioral responses to noise and silence were assessed. ABRs were measured subsequently (ABR 3). The difference between ABR 1 and ABR 3 was considered the one-day threshold shift (purple).

**Figure 3:**
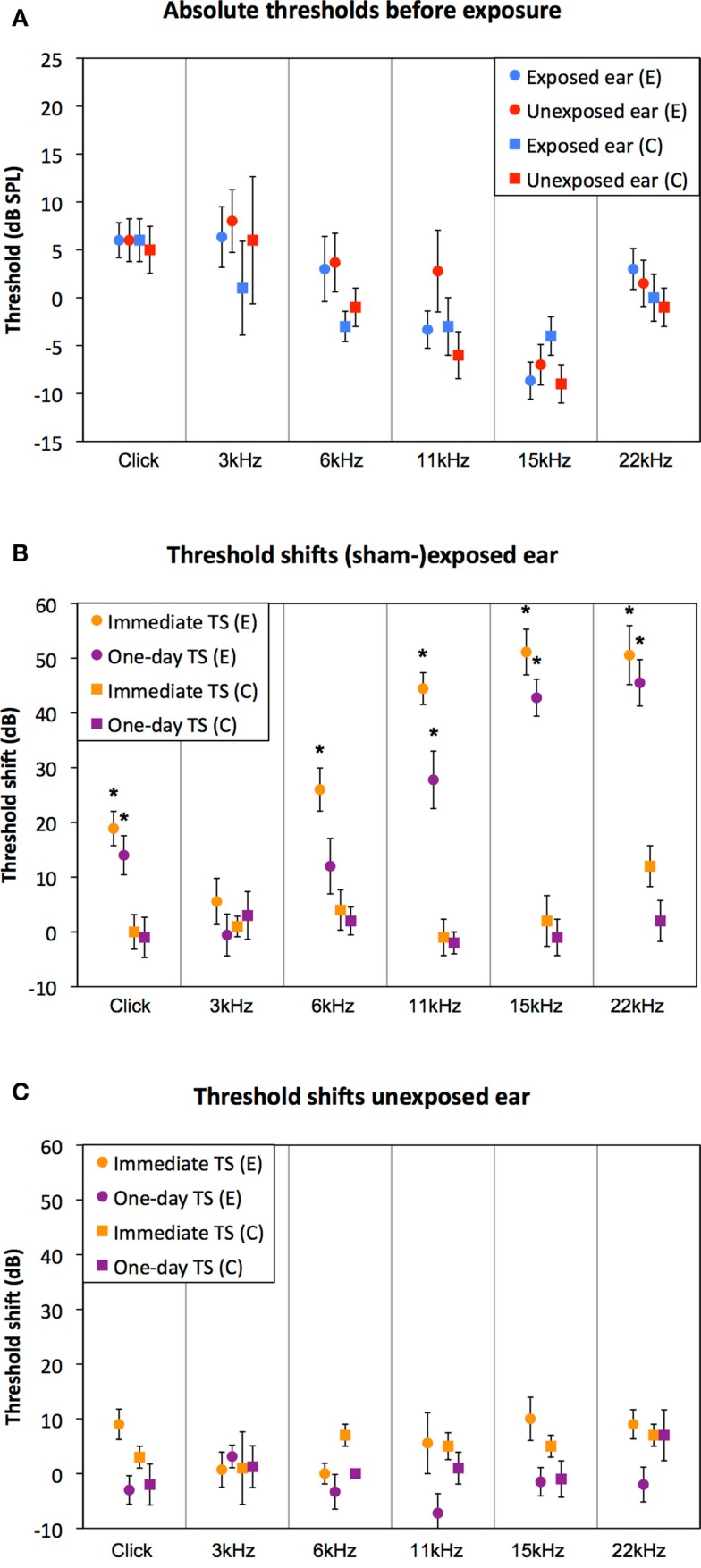
**Auditory thresholds and sound-induced threshold shifts are shown**. Absolute hearing thresholds before exposure were similar between the control group (squared markers) and the experimental group (circled markers). Before exposure, there were no differences between the two ears in both groups (red vs. blue markers) (**A**). Threshold shifts immediately (orange curves) and 1 day (purple curves) after overexposure in the exposed ear **(B)** and the unexposed ear **(C)** of the experimental group (circled markers) and the control group (squared markers). * indicates a significant threshold shift relative to ABRs before exposure (ABR 1; *T*-tests Bonferroni corrected, *p* < 0.05).

Auditory thresholds assessed before exposure (ABR 1 in Figure 2) were similar between the control and the experimental group, for all measured stimuli (Figure 3A, RM-ANOVA: n.s.). Immediately following exposure (ABR 2 in Figure 2), thresholds of the exposed ear of the experimental group were elevated for all stimuli, except 3 kHz [Figure 3B, orange circles; RM-ANOVA: *F*(5,80) = 25.125, *p* < 0.001, Bonferroni corrected paired *T*-tests, *p* < 0.05]. One day after exposure (ABR 3 in Figure 2), thresholds partly recovered, but were still significantly elevated, except for 3 and 6 kHz [Figure 3B, purple circles; RM-ANOVA: *F*(5,85) = 18.426, *p* < 0.001, Bonferroni corrected paired *T*-tests, *p* < 0.05]. Thresholds of the sham-exposed ears were not affected (Figure 3B, squares; RM-ANOVA: n.s.). In addition, unilateral (sham-) exposure did not affect the auditory thresholds of the unexposed ear (Figure 3C; RM-ANOVA: n.s.), indicating that the unilateral exposure only affected the ipsilateral ear. Accordingly, all animals were able to hear the stimulus with at least one ear at the day of behavioral testing.

**Figure 4:**
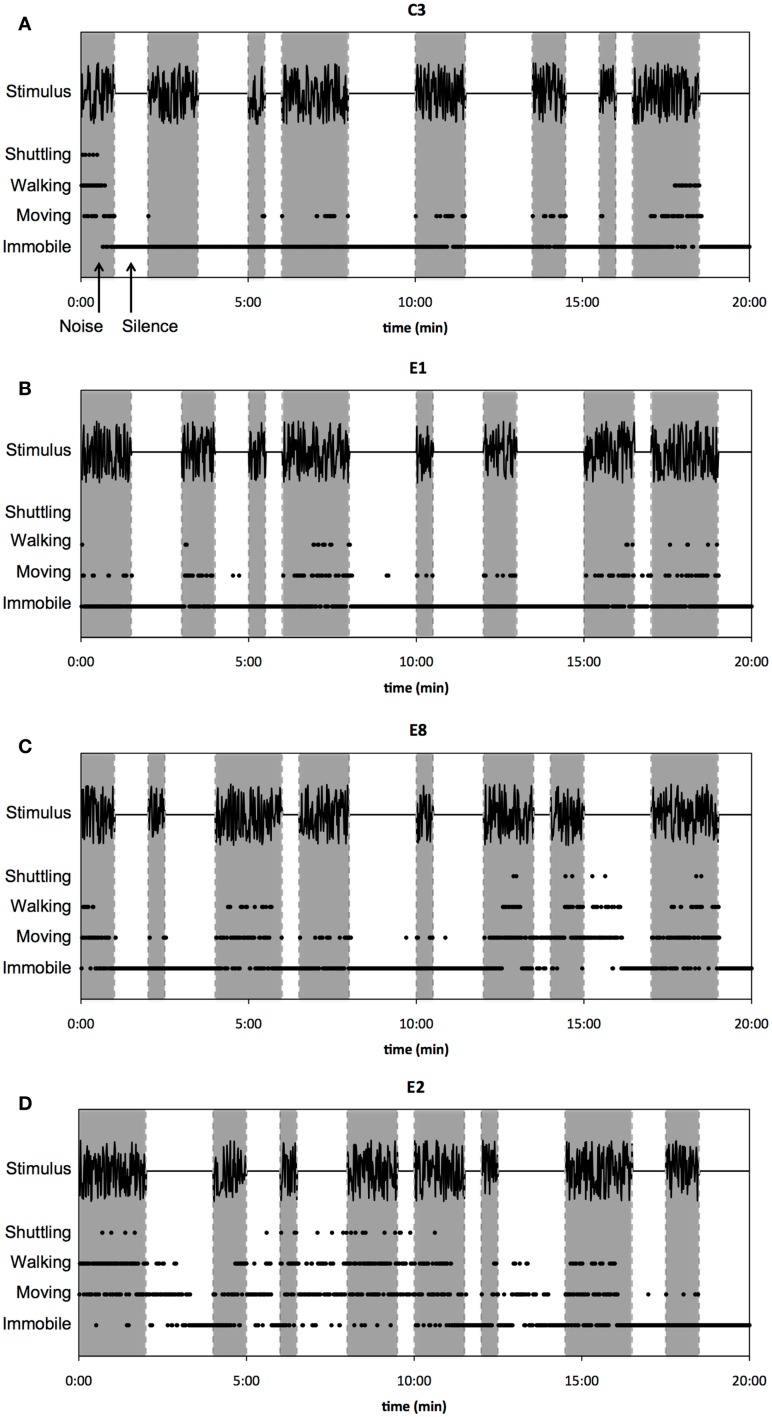
**Raw data of spontaneous behavior in noise and silence is shown**. Examples of behavior during noise (shaded timeframes) and silence (unshaded timeframes) of a control animal **(A)**, an exposed animal classified as “unaffected” **(B)**, and two exposed animals classified as “affected” [**(C,D)**, respectively]. Every dot represents 1 s; every second was classified for either immobile, moving, walking, or shuttling (see Materials and Methods, section “Testing of behavioral activity in noise and silence”). As can be seen, the two animals classified as “affected” **(C,D)** were more active during silent intervals compared to the control **(A)** and the “unaffected” animal **(B)**.

The (normal-hearing) control animals were predominantly immobile during silent intervals, as can be seen in the representative example of spontaneous behavior of a sham-exposed guinea pig (C3) during noise and silence intervals (Figure 4A; noise intervals are indicated with shaded columns, silence intervals with white columns). This animal was immobile during silence for 99.5% of the time (597 of 600 s). During noise intervals, guinea pig C3 was immobile for only 73% of the time (438 of 600 s) and engaged in the other scored behaviors for the remaining time, i.e., shuttle (1%), walking (8%), and moving (18%). Studying spontaneous behavior of the sound-exposed animals, however, revealed that immobility during silence varied more in the sound-exposed group compared to the sham-exposed group. Figure 4B displays a representative example of a guinea pig (E1) that showed spontaneous behavior that was similar to the control animals. This animal was immobile during silence for 97.2% of the time (583 of 600 s). In noise, guinea pig E1 was immobile for only 75.8% of the time (455 of 600 s) and was moving and walking for 20% and 4.2% of the time, respectively. Guinea pig E1 did not shuttle in any interval of the stimulus. On the contrary, guinea pig E8 became less immobile in the silent intervals after approximately 10 min of behavioral testing. On average, this animal was immobile during silence for 84.2% of the time (505 of 600 s; Figure 4C). Additionally, guinea pig E8 shuttled six times during the recorded time, of which twice during a silent interval. Furthermore, guinea pig E2 was relatively active during most of the silent intervals and was immobile for 57.8% of the time in silence (347 of 600 s). This animal shuttled 22 times in total, of which 8 times in the silent intervals (Figure 4D).

**Figure 5:**
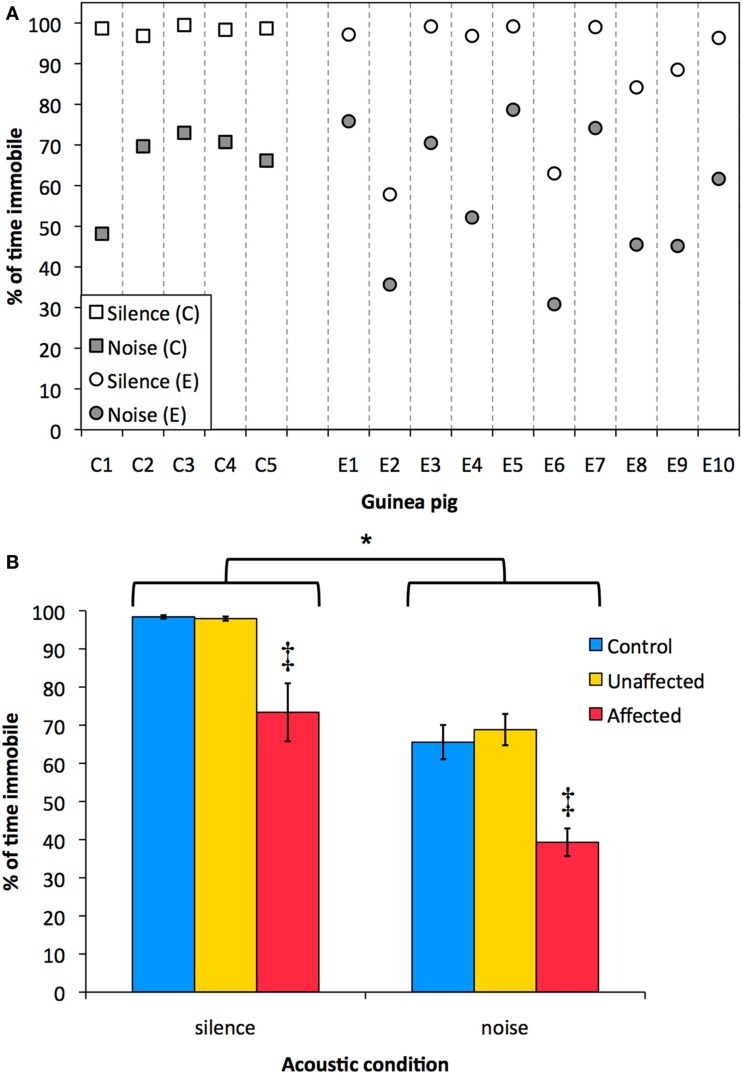
**Immobility in noise and silence is shown**. **(A)** Individual immobility (% of time) in silence (white markers) and in noise (gray markers) for the control group (C1–C5; squares) and the exposed group (E1–E10; circles). **(B)** Mean immobility (% of time) ± SEM during noise and during silence for the control group (blue), the “unaffected” group (yellow), and the “affected” group (red). There was a significant difference between immobility in noise and in silence [*, RM-ANOVA: *F*(1,12) = 164.7, *p* < 0.001]. Furthermore, the “affected” group was significantly less immobile than the control group and the “unaffected” group, both in silence and in noise [‡, RM-ANOVA: *F*(2,12) = 20.06, *p* < 0.001, Bonferroni corrected pair-wise comparisons].

Exposed guinea pigs were divided between an “affected” and an “unaffected” group based on their immobility in silence. The mean immobility in silence (98.4%) minus three times the SD of the control group (3 × 0.98%) was the criterion above which an animal of the exposed group was classified as “unaffected.” When the immobility in silence of an exposed animal was lower than the criterion, it was classified as an “affected” animal. Six of the 10 exposed guinea pigs (E1, E3, E4, E5, E7, and E10) showed similar behavior during silence as the control animals (>95.5% immobility in silence) and were classified as “unaffected.” The remaining four exposed guinea pigs (E2, E6, E8, and E9) had a immobility in silence lower than the 95.5%, and were classified as “affected” (Figure 5A). See Supplementary Material for a visual example of spontaneous behavior in noise and silence of a control animal (C2), an animal classified as “unaffected” (E4), and an animals classified as “affected” (E6).

Sham-exposed animals were significantly longer immobile during silence than during noise [Figure 5B, blue bars; paired-sample *T*-test: *T*(4) = 7.240, *p* < 0.005]. Furthermore, sham-exposed animals shuttled on average eight times during noise intervals (range 5–11), yet, they never shuttled in the silent intervals. The percentage immobility of the “affected” group was significantly different from both the control group and the “unaffected” group, both in silence and in noise [Figure 5B; RM-ANOVA: *F*(2,12) = 20.06, *p* < 0.001, Bonferroni corrected pair-wise comparisons]. The effect of acoustic condition on immobility was significant [RM-ANOVA: *F*(1,12) = 164.7, *p* < 0.001] and there was no interaction between acoustic condition and the groups (RM-ANOVA: *p* > 0.68).

## Discussion

The present data demonstrate that guinea pigs can be trained relatively easy to shuttle in response to an acoustic CS. However, training to shuttle in response to silent intervals in noise was unsuccessful (experiment 1). We showed in experiment 2 that normal-hearing guinea pigs hardly moved during silence, while they frequently moved during presentation of noise. This natural tendency for immobility during silence may explain the failure to train guinea pigs to silence in experiment 1. Furthermore, unilateral exposure to a loud sound led to a decrease in immobility during silence in a subset of the exposed animals. This might suggest that these exposed animals (labeled as “affected”) no longer experienced silence: they may have perceived tinnitus. However, these animals were also more active in noise compared to the control animals. Therefore, it is not clear whether the “affected” animals were under stimulus control, i.e., the increased activity in both silence and noise could have been the result from additional factors other than the presented acoustic stimuli. These additional factors might include tinnitus, but can also involve, for example, an overall increase in general activity or anxiety. Accordingly, conducting several validation experiments is crucial before this method can be reliably used as a screening tool for tinnitus in guinea pigs. These necessary validation experiments are extensively discussed below and should further elucidate the origin of the abnormal behavior of these animals.

By showing that guinea pigs can be trained within 10 days to respond with a shuttle response to a noise interval, we confirmed earlier studies with the shuttle box (14, 17, 19). It has been shown that guinea pigs can generalize this response to other sound levels and to intracochlear electrical stimulation (17, 19). Guinea pigs can also be trained to respond to pure tones, although in different training paradigms (21–25). In spite of these positive findings, training guinea pigs to respond to silent intervals (CS) in noise was unsuccessful, both in naïve and previously trained animals. Instead, during experiment 1, guinea pigs seemed to become immobile at the onset of silence. This might explain why a successful conditioning model to test for tinnitus in guinea pigs has not been reported in literature yet.

Experiment 2 confirmed that, indeed, normal-hearing guinea pigs demonstrated significantly more immobility in silence than in noise. Moreover, normal-hearing guinea pigs did not demonstrate a single spontaneous shuttle response during a silent interval, while during noise, they demonstrated on average eight spontaneous shuttle responses in 10 min. The mobility during a noise interval might have been crucial for successful training in the shuttle box in experiment 1, and explains why training to silent intervals was unsuccessful.

Furthermore, in experiment 2, the guinea pigs in the experimental group were unilaterally exposed for 1 h to an 11-kHz tone of 124 dB SPL. This type of acoustic trauma has previously been proposed to induce tinnitus in guinea pigs (12). Our results showed that a subset of the exposed animals, classified as “affected,” were more active both in noise and in silence (see Figure 5B). This finding can be due to several factors, which are described in the following paragraphs.

For example, natural variation in general activity might be an explanation for this behavior. A natural variation in anxiety and exploratory behavior is apparent in mice and rats (26, 27). Therefore, it is conceivable that the guinea pigs in the “affected” group were more active by nature, resulting in less immobility in both silence and noise. However, the finding that normal-hearing animals hardly moved in silence was a robust result, as reflected by the failed training in experiment 1, the control group of experiment 2, several pilot experiments with normal-hearing guinea pigs in our lab (data not shown), and by the literature (19). Specifically, in the control group of experiment 2, the mean immobility rate was 98.4% with a SD of 0.98%. The immobility of the four animals classified as “affected” was between 10 and 42 SD from the mean of the control group. Therefore, it is unlikely that the increased mobility of the affected guinea pigs is caused by natural variation across all animals.

Another factor that could be involved in the observed increase in behavioral activity in noise and silence is the hearing loss induced by the sound exposure. Previous research has shown that hearing loss affects the startle response (28). However, the startle response is a reflex. In contrast, the behavior of the current study is likely to be not a reflex, since it is expressed as long as at least 120 s. Moreover, note that both the “affected” and the “unaffected” animals in the exposed group had a unilateral hearing loss. Thus, the presence of unilateral hearing loss can probably not explain the abnormal behavior in the “affected” group.

One could argue that the observed behavior in the “affected” group is a result of stress and anxiety, since numerous interactions between the auditory and limbic system have been demonstrated (29). In the current study, all animals were under anesthesia during the (sham) exposure. Thus, the stress induced by the procedure is being controlled for, and the actual traumatizing noise is most likely not experienced by the exposed animals. Any stress that the exposed animals might have, must have derived from damage due to the noise exposure. For example, noise trauma, applied under anesthesia, can affect brain structures, such as the neurogenesis in the hippocampus (30), which would presumably affect behavior. Indeed, Zheng and colleagues found that tinnitus-inducing noise exposure impairs several instinctive behaviors, such as impulsive control and social interaction, but not anxiety (31, 32). Moreover, if the “affected” animals were more anxious, they would presumably show a decrease, rather than an increase, in activity due to freezing behavior, which is a well-established measure for anxiety (33–35). Thus, stress and anxiety can probably not account for the increased mobility of the animals in the “affected” group.

Another explanation for the abnormal behavior in the “affected” animals is that they perceived tinnitus. Namely, by interpreting immobility as a signature of perceiving silence, the presence of tinnitus would decrease the amount of immobility in silence, because the animals might not detect complete silence anymore. As such, tinnitus would function as a masker of the silence. Therefore, one possible explanation for the increased activity in silence is that these animals perceived tinnitus that masked the silent intervals. Similar to earlier studies demonstrating that unilateral trauma leads to tinnitus in only a subset of guinea pigs (11, 20), not all exposed animals demonstrated this increased activity during silence. This finding is also consistent with the observation that acoustic trauma in humans does not lead to tinnitus in all human subjects (36, 37). Previous psychophysical studies in humans suggest that tinnitus does not mask the offset of sound; listeners with tinnitus can still detect silent gaps in noise (38, 39). Thus, the increased activity in silence of the exposed “affected” animals is rather related to the inability to detect complete silence than to an inability to detect the offset of the noise. There were different degrees of increased activity in silence within animals labeled as “affected.” This could reflect that this test is more sensitive for one animal with tinnitus than for the other. However, it could also reflect different characteristics or intensities of tinnitus. The latter is reasonable, because there are considerable inter-individual differences in the characteristics of tinnitus induced by noise exposure, both in humans and in laboratory animals (20, 37). The increased mobility in noise could be explained by the presence of hyperacusis, i.e., an oversensitivity to sound, which is reported to be often comorbid with tinnitus (40). This could have resulted in a more intense perception of the already quite loud noise stimulus of 88 dB SPL. Thus, the range of behaviors observed in the “affected” animals could reflect the range of tinnitus characteristics.

However, validation experiments are crucial before the abnormal behavior can be attributed to tinnitus. The fact that the control and the “unaffected” animals show a different behavior in noise and silence, respectively, shows that they are most likely under control of the acoustic stimulus (41). However, the “affected” animals are (at least) partly not under stimulus control, since they are mobile in silence but also show increased activity in noise. Above, we hypothesized that the perception of tinnitus can explain the increased activity in silence. However, numerous confounding effects of noise exposure could have also controlled their behavior, such as unilateral hearing loss, hyperacusis, motoric changes, generalized hyperactivity, stress, and anxiety. The following validation experiments are proposed to confirm or deny our hypothesis that the abnormal behavior of the affected group is due to tinnitus (42–44):

1. Measuring corticosterone levels before behavioral testing can determine whether there is a correlation between stress and spontaneous behavior in noise and silence.
2. Inducing stress in normal-hearing animals before behavioral testing may show the effects of stress and anxiety on the behavior of guinea pigs in noise and silence.
3. An ear-plugging experiment, which is also used to validate other tinnitus behavioral models (28, 45, 46), can reveal whether unilateral conductive hearing loss affects immobility in silence. However, one should interpret the outcomes with caution, since conductive hearing loss may also induce tinnitus (47).
4. If the increased activity in both noise and silence of the “affected” animals is due to a generalized increase in activity, it should be independent of the acoustic characteristics of the noise. The increased activity should be observed for both weak and strong noise stimuli. Instead, if the increased activity during noise is caused by hyperacusis, it would disappear when softer stimuli are used. Thus, repeating these experiments with weaker stimuli might test whether the abnormal behavior is due to an generalized increase in activity or due to hyperacusis (and tinnitus during the silent intervals).
5. In a within-subject design, spontaneous behavior could be assessed before and after sound exposure. This could test for a contribution of a natural variation in general activity as an explanation for the differences between the “affected” and “unaffected” animals.
6. To determine whether the behavior of the “affected” animals could be due to the perception of a sound (i.e., tinnitus), a tinnitus-like sound can be presented to a normal-hearing guinea pig during the entire testing period of the current paradigm (48). Behavior in silence and noise that is similar to that of the “affected” animals supports the hypothesis that this behavior is due to the perception of a sound, which would be tinnitus in the “affected” animals.
7. Testing other tinnitus-inducing agents, such as salicylate, for their effect on the behavior of guinea pigs in noise and silence can also reveal whether the observed behavior in the “affected” group could be a result of tinnitus (49). In this experiment, one should keep in mind that certain drugs might also have their own effect directly on behavior, independently from their effect on the auditory system.
8. Cross-validation against other behavioral models to test for tinnitus, for example, the startle reflex paradigm, may provide further validation (13).
9. It would further validate our measure when neurophysiological changes that are often attributed to tinnitus are found in the “affected” but not in the “unaffected” animals (7–10).

As discussed previously, current behavioral animal models for tinnitus can be divided in two categories: (1) conditioning paradigms (5) and (2) startle reflex paradigms (6). If the above-described phenomenon does indeed represent tinnitus in guinea pigs, it would be adding a new category to the existing behavioral tinnitus models. An advantage of the current model is that no training is required, which allows efficient assessment in large groups of animals. Furthermore, if the observed behavior is indeed instinctive, as expected, aging and other factors that influence learning and memory are not likely to affect outcomes.

In conclusion, guinea pigs could be trained relatively easy to detect noise in an active avoidance task, but did not show this trained response to silent intervals in continuous noise (experiment 1). Apparent immobility during silent intervals explains this finding (experiment 2). Further, we showed that a subset of noise-exposed animals was less immobile during silence and noise. We suggested that this abnormal behavior may be due to tinnitus and hyperacusis, respectively. However, conducting several validation experiments is very important before implementing this method as a new measure to detect tinnitus. Therefore, a number of experiments have been proposed that may further elucidate the origin of the abnormal behavior.

## Conflict of Interest Statement

The authors declare that the research was conducted in the absence of any commercial or financial relationships that could be construed as a potential conflict of interest.

## Supplementary Material

The Supplementary Material for this article can be found online at http://www.frontiersin.org/Journal/10.3389/fneur.2014.00207/abstract

## Abbreviations

ABR, auditory brainstem response; CAR, conditioned avoidance response; CS, conditioned stimulus; RM-ANOVA, repeated-measures analysis of variance; UCS, unconditioned stimulus.

## References

[1] Salvi RJ Saunders SS Gratton MA Arehole S Powers N Enhanced evoked response amplitudes in the inferior colliculus of the chinchilla following acoustic trauma. Hear Res (1990) 50:245–58 10.1016/0378-5955(90)90049-U 2076976

[2] Noreña AJ Eggermont JJ Changes in spontaneous neural activity immediately after an acoustic trauma: implications for neural correlates of tinnitus. Hear Res (2003) 183:137–53 10.1016/S0378-5955(03)00225-9 13679145

[3] Shore SE Koehler S Oldakowski M Hughes LF Syed S Dorsal cochlear nucleus responses to somatosensory stimulation are enhanced after noise-induced hearing loss. Eur J Neurosci (2008) 27:155–68 10.1111/j.1460-9568.2007.05983.x 18184319 PMC2614620

[4] Turner JG Behavioral measures of tinnitus in laboratory animals. Prog Brain Res (2007) 166:147–56 10.1016/S0079-6123(07)66013-0 17956779

[5] Jastreboff PJ Brennan JF Sasaki CT An animal model for tinnitus. Laryngoscope (1988) 98:280–6 10.1288/00005537-198803000-00008 2830445

[6] Turner JG Brozoski TJ Bauer CA Parrish JL Myers K Hughes LF Gap detection deficits in rats with tinnitus: a potential novel screening tool. Behav Neurosci (2006) 120(1):188–95 10.1037/0735-7044.120.1.188 16492129

[7] Jastreboff PJ Sasaki CT Salicylate-induced changes in spontaneous activity of single units in the inferior colliculus of the guinea pig. J Acoust Soc Am (1986) 80(5):1384–91 10.1121/1.394391 3782617

[8] Takemura K Komeda M Yagi M Himeno C Izumikawa M Doi T Direct inner ear infusion of dexamethasone attenuates noise-induced trauma in guinea pig. Hear Res (2004) 196:58–68 10.1016/j.heares.2004.06.003 15464302

[9] Noreña AJ Moffat G Blanc JL Pezard L Cazals Y Neural changes in the auditory cortex of awake guinea pigs after two tinnitus inducers: salicylate and acoustic trauma. Neuroscience (2010) 166:1194–209 10.1016/j.neuroscience.2009.12.063 20096752

[10] Mulders WHAM Ding D Salvi R Robertson D Relationship between auditory thresholds, central spontaneous activity, and hair cell loss after acoustic trauma. J Comp Neurol (2011) 519:2637–47 10.1002/cne.22644 21491427 PMC3140598

[11] Dehmel S Eisinger D Shore SE Gap prepulse inhibition and auditory brainstem-evoked potentials as objective measures for tinnitus in guinea pigs. Front Syst Neurosci (2012) 6:42 10.3389/fnsys.2012.00042 22666193 PMC3364697

[12] Robertson D Bester C Vogler D Mulders WHAM Spontaneous hyperactivity in the auditory midbrain: relationship to afferent input. Hear Res (2013) 295:124–9 10.1016/j.heares.2012.02.002 22349094

[13] Berger JI Coomber B Shackleton TM Palmer AR Wallace MN A novel behavioural approach to detecting tinnitus in the guinea pig. J Neurosci Methods (2013) 213:188–95 10.1016/j.jneumeth.2012.12.023 23291084 PMC3580292

[14] Philippens IHCHM Melchers BPC Wolthuis OL Active avoidance behavior in guinea pigs: effects of physostigmine and scopolamine. Pharmacol Biochem Behav (1992) 42:285–9 10.1016/0091-3057(92)90528-N 1631181

[15] Diamond IT Neff WD Ablation of temporal cortex and discrimination of auditory patterns. J Neurophysiol (1957) 20(3):300–15 13429392 10.1152/jn.1957.20.3.300

[16] Böhmer A The Preyer reflex – an easy estimate of hearing function in guinea pigs. Acta Otolaryngol (1988) 106:368–72 10.3109/00016488809122259 3207004

[17] Agterberg MJH van den Broek M Philippens IHCHM A less stressful animal model: a conditioned avoidance behaviour task for guinea pigs. Lab Anim (2010) 44:206–10 10.1258/la.2009.009096 20071411

[18] Sansone M Bovet D Avoidance learning by Guinea pigs. Q J Exp Psychol (1970) 22(3):458–61 10.1080/14640747008401919

[19] Agterberg MJ Versnel H Behavioral responses of deafened guinea pigs to intracochlear electrical stimulation: a new rapid psychophysical procedure. Hear Res (2014) 313:67–74 10.1016/j.heares.2014.04.011 24811981

[20] Coomber B Berger JI Kowalkowski VL Shackleton TM Palmer AR Wallace MN Neural changes accompanying tinnitus following unilateral acoustic trauma in the guinea pig. Eur J Neurosci (2014) 40(2):2427–41 10.1111/ejn.12580 24702651 PMC4215599

[21] Crifò S Shiver-audiometry in the conditioned guinea-pig (simplified Anderson-Wedenberg test). Acta Otolaryngol (1973) 75:38–44 10.3109/00016487309139636 4689027

[22] Prosen CA Petersen MR Moody DB Stebbins WC Auditory thresholds and kanamycin-induced hearing loss in the guinea pig assessed by a positive reinforcement procedure. J Acoust Soc Am (1978) 63(2):559–66 10.1121/1.381754 670552

[23] Bakin JS South DA Weinberger NM Induction of receptive field plasticity in the auditory cortex of the guinea pig during instrumental avoidance conditioning. Behav Neurosci (1996) 110(5):905–13 10.1037/0735-7044.110.5.905 8918994

[24] Galván VV Weinberger NM Long-term consolidation and retention of learning-induced tuning plasticity in the auditory cortex of the guinea pig. Neurobiol Learn Mem (2002) 77:78–108 10.1006/nlme.2001.4044 11749087

[25] Hu B Lin X Huang L-S Yang L Feng H Sui J-F Involvement of the ipsilateral and contralateral cerebellum in the acquisition of unilateral classical eyeblink conditioning in guinea pigs. Acta Pharmacol Sin (2009) 30(2):141–52 10.1038/aps.2008.18 19122670 PMC4002461

[26] Kazlauckas V Schuh J Dall’Igna OP Pereira GS Bonan CD Lara DR Behavioral and cognitive profile of mice with high and low exploratory phenotypes. Behav Brain Res (2005) 162(2):272–8 10.1016/j.bbr.2005.03.021 15970221

[27] Herrero AI Sandi C Venero C Individual differences in anxiety trait are related to spatial learning abilities and hippocampal expression of mineralocorticoid receptors. Neurobiol Learn Mem (2006) 86(2):150–9 10.1016/j.nlm.2006.02.001 16580234

[28] Lobarinas E Hayes SH Allman BL The gap-startle paradigm for tinnitus screening in animal models: limitations and optimization. Hear Res (2013) 295:150–60 10.1016/j.heares.2012.06.001 22728305 PMC3505812

[29] Kraus KS Canlon B Neuronal connectivity and interactions between the auditory and limbic systems. Effects of noise and tinnitus. Hear Res (2012) 288:34–46 10.1016/j.heares.2012.02.009 22440225

[30] Kraus KS Mitra S Jimenez Z Hinduja S Ding D Jiang H Noise trauma impairs neurogenesis in the rat hippocampus. Neuroscience (2010) 167(4):1216–26 10.1016/j.neuroscience.2010.02.071 20206235 PMC2952397

[31] Zheng Y Hamilton E Stiles L McNamara E de Waele C Smith PF Acoustic trauma that can cause tinnitus impairs impulsive control but not performance accuracy in the 5-choice serial reaction time task in rats. Neuroscience (2011) 180:75–84 10.1016/j.neuroscience.2011.02.040 21352899

[32] Zheng Y Hamilton E McNamara E Smith PF Darlington CL The effects of chronic tinnitus caused by acoustic trauma on social behaviour and anxiety in rats. Neuroscience (2011) 193:143–53 10.1016/j.neuroscience.2011.07.026 21782007

[33] Crawford M Masterson FA Species-specific defense reactions and avoidance learning. Pavlov J Biol Sci (1982) 17(4):204–14 6891452 10.1007/BF03001275

[34] Rodgers RJ Animal models of ‘anxiety’: where next? Behav Pharmacol (1997) 8(6–7):477–96 10.1097/00008877-199711000-00003 9832964

[35] Hagenaars MA Oitzl M Roelofs K Updating freeze: aligning animal and human research. Neurosci Biobehav Rev (2014) 47C:165–76 10.1016/j.neubiorev.2014.07.021 25108035

[36] Kitcher ED Ocansey G Tumpi DA Early occupational hearing loss of workers in a stone crushing industry: our experience in a developing country. Noise Health (2012) 14(57):68–71 10.4103/1463-1741.95134 22517306

[37] Degeest S Corthals P Vinck B Keppler H Prevalence and characteristics of tinnitus after leisure noise exposure in young adults. Noise Health (2014) 16(68):26–33 10.4103/1463-1741.127850 24583677

[38] Campolo J Lobarinas E Salvi R Does tinnitus “fill in” the silent gaps? Noise Health (2013) 15(67):398–405 10.4103/1463-1741.121232 24231418 PMC3875329

[39] Fournier P Hébert S Gap detection deficits in humans with tinnitus as assessed with the acoustic startle paradigm: does tinnitus fill in the gap? Hear Res (2013) 295:16–23 10.1016/j.heares.2012.05.011 22688322

[40] Schecklmann M Landgrebe M Langguth B the TRI Database Study Group Phenotypic characteristics of hyperacusis in tinnitus. PLoS One (2014) 9(1):e86944 10.1371/journal.pone.0086944 24498000 PMC3908961

[41] Sidman M Reflections on stimulus control. Behav Anal (2008) 31(2):127–35 22478506 10.1007/BF03392166 PMC2591753

[42] Heffner HE Harrington IA Tinnitus in hamsters following exposure to intense sound. Hear Res (2002) 170:83–95 10.1016/S0378-5955(02)00343-X 12208543

[43] Heffner HE Heffner RS Behavioral tests for tinnitus in animals. In: Eggermont JJ, editor. Tinnitus. New York: Springer Science+Business Media (2012). p. 21–58

[44] von der Behrens W Animal models of subjective tinnitus. Neural Plast (2014) 2014:741452 10.1155/2014/741452 24829805 PMC4009209

[45] Heffner HE Koay G Tinnitus and hearing loss in hamsters (*Mesocricetis auratus*) exposed to loud sound. Behav Neurosci (2005) 119(3):734–42 10.1037/0735-7044.119.3.734 15998194

[46] Bauer CA Brozoski TJ Assessing tinnitus and prospective tinnitus therapeutics using a psychophysical animal model. J Assoc Res Otolaryngol (2001) 2(1):54–64 11545150 10.1007/s101620010030 PMC3201094

[47] Mills RP Cherry JR Subjective tinnitus in children with otological disorders. Int J Pediatr Otorhinolaryngol (1984) 7(1):21–7 10.1016/S0165-5876(84)80050-6 6539313

[48] Jastreboff PJ Brennan JF Coleman JK Sasaki CT Phantom auditory sensation in rats: an animal model for tinnitus. Behav Neurosci (1988) 102(6):811–22 10.1037/0735-7044.102.6.811 3214530

[49] Guitton MJ Caston J Ruel J Johnson RM Pujol R Puel J-L Salicylate induces tinnitus through activation of cochlear NMDA receptors. J Neurosci (2003) 23(9):3944–52 12736364 10.1523/JNEUROSCI.23-09-03944.2003 PMC6742173

